# Supplementary material for: Removal of Antibiotic Resistance Genes, Class 1 Integrase Gene and Escherichia coli Indicator Gene in a Microalgae-Based Wastewater Treatment System
Source: Antibiotics (Basel). 2022 Nov 2;11(11):1531. doi: 10.3390/antibiotics11111531 (PMC9686833; doi:10.3390/antibiotics11111531)
Supplement: Supplementary file 1 [file antibiotics-11-01531-s001.zip › antibiotics-1969347-supplementary.pdf]

Supplementary

Removal of Antibiotic Resistance Genes, Class 1 Integrase Gene and *Escherichia coli* Indicator Gene in a Microalgae-Based Wastewater Treatment System

Table S1 Primer pairs and annealing temperatures used in the current study.

Primer	Primer sequence (5′-3′)	Annealing Temp./Number of cycles	Reference
338-F<br>518-R	ACTCCTACGGGAGGCAGCAG<br>ATTACCGCGGCTGCTGG	58°C/35	Yan et al. (2018)
intI1-F<br>intI1-R	GGCTTCGTGATGCCTGCTT<br>CATTCCTGGCCGTGGTTCT	55°C/35	Chen et al. (2016)
tetO-F<br>tetO-R	ACGGARAGTTTATTGTATACC<br>TGGCGTATCTATAATGTTGAC	55°C/35	Al-Jassim et al. (2015)
tetW-F<br>tetW-R	GAGAGCCTGCTATATGCCAGC<br>GGGCGTATCCACAATGTTAAC	60°C/40	Al-Jassim et al. (2015)
tetX-F<br>tetX-R	AGCCTTACCAATGGGTGTAAA<br>TTCTTACCTTGGACATCCCG	60°C/40	Dong et al. (2019)
bla_{TEM}-F<br>bla_{TEM}-R	AGCATCTTACGGATGGCATGA<br>TCCTCCGATCGTTGTCAGAAGT	60°C /40	Gao et al. (2020)
bla_{CTX}-F<br>bla_{CTX}-R	AGTGAAAGCGAACCGAATC<br>CTGTCACCAATGCTTTACC	55°C/40	Wen et al. (2016)
ermB-F<br>ermB-R	AAAACTTACCCGCCATACCA<br>TTTGGCGTGTTTCATTGCTT	55°C/40	Knapp et al. (2010)
aadA-F<br>aadA-R	GTTGTGCACGACGACATCAT<br>TGGTGGTACTTCATCGGCATA	55°C/35	Su et al. (2020)
sul1-F<br>sul1-R	CACCGGAAACATCGCTGCA<br>AAGTTCCGCCGCAAGGCT	55°C/35	Cheng et al. (2020)
sul2-F<br>sul2-R	GCGCTCAAGGCAGATGGCATT<br>GCGTTTGATACCGGCACCCGT	55°C/40	Cheng et al. (2020)
cmlA-F<br>cmlA-R	GCCAGCAGTGCCGTTTAT<br>GGCCACCTCCCAGTAGAA	55°C/40	Chen et al. (2016)
floR-F<br>floR-R	CGGTCGGTATTGTCTTCACG<br>TCACGGGCCACGCTGTAT	58°C/40	Chen et al. (2016)
uidA-F<br>uidA-R	AAAACGGCAAGAAAAAGCAG<br>ACGCGTGGTTACAGTCTTGCG	60°C/40	Adekanmbi et al. (2020)

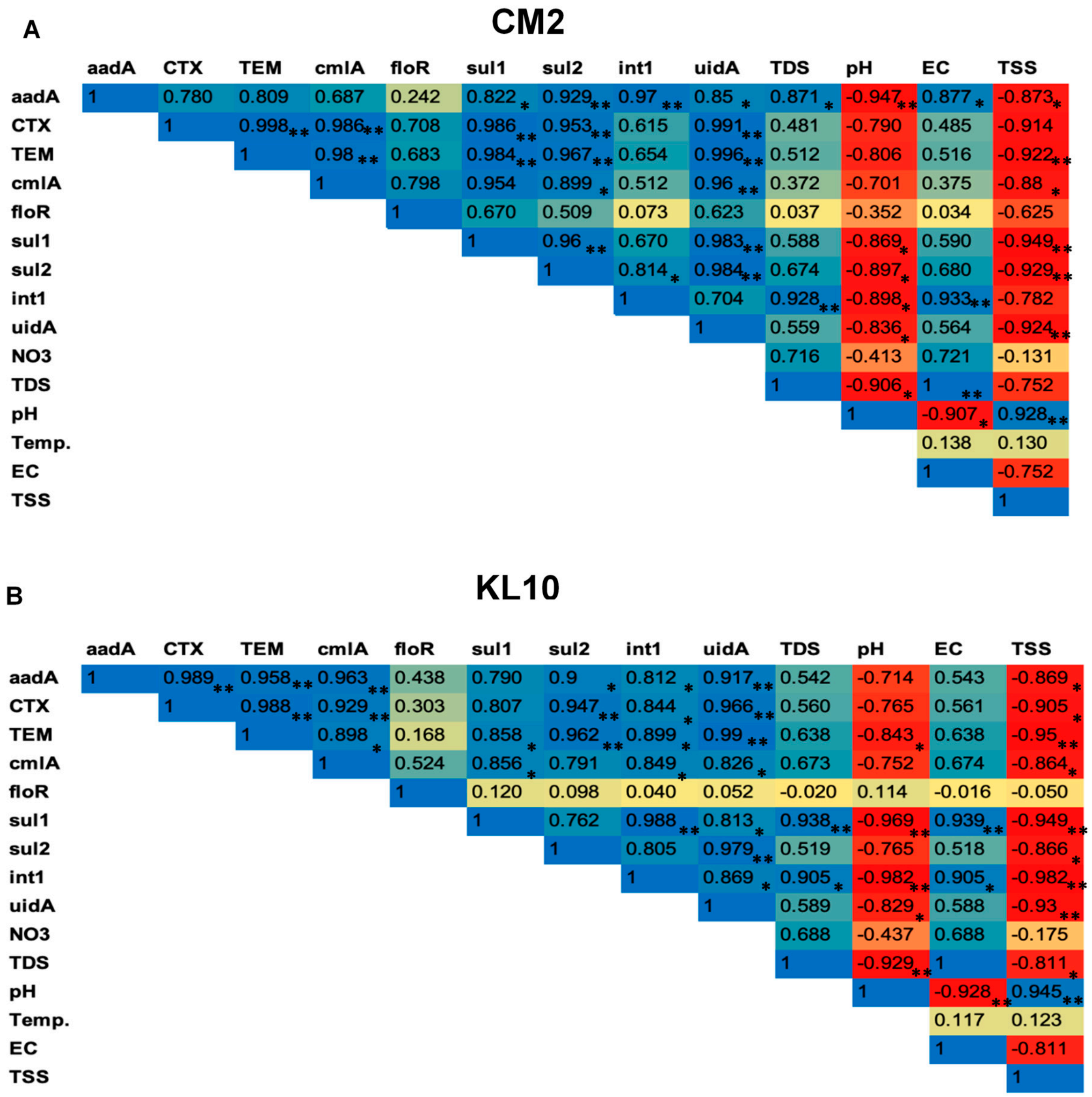

Figure S1. Correlation of the study genes with physicochemical water quality parameters in the monocultures. ** Correlation is significant at the 0.01 level (2-tailed); *. Correlation is significant at the 0.05 level (2-tailed).

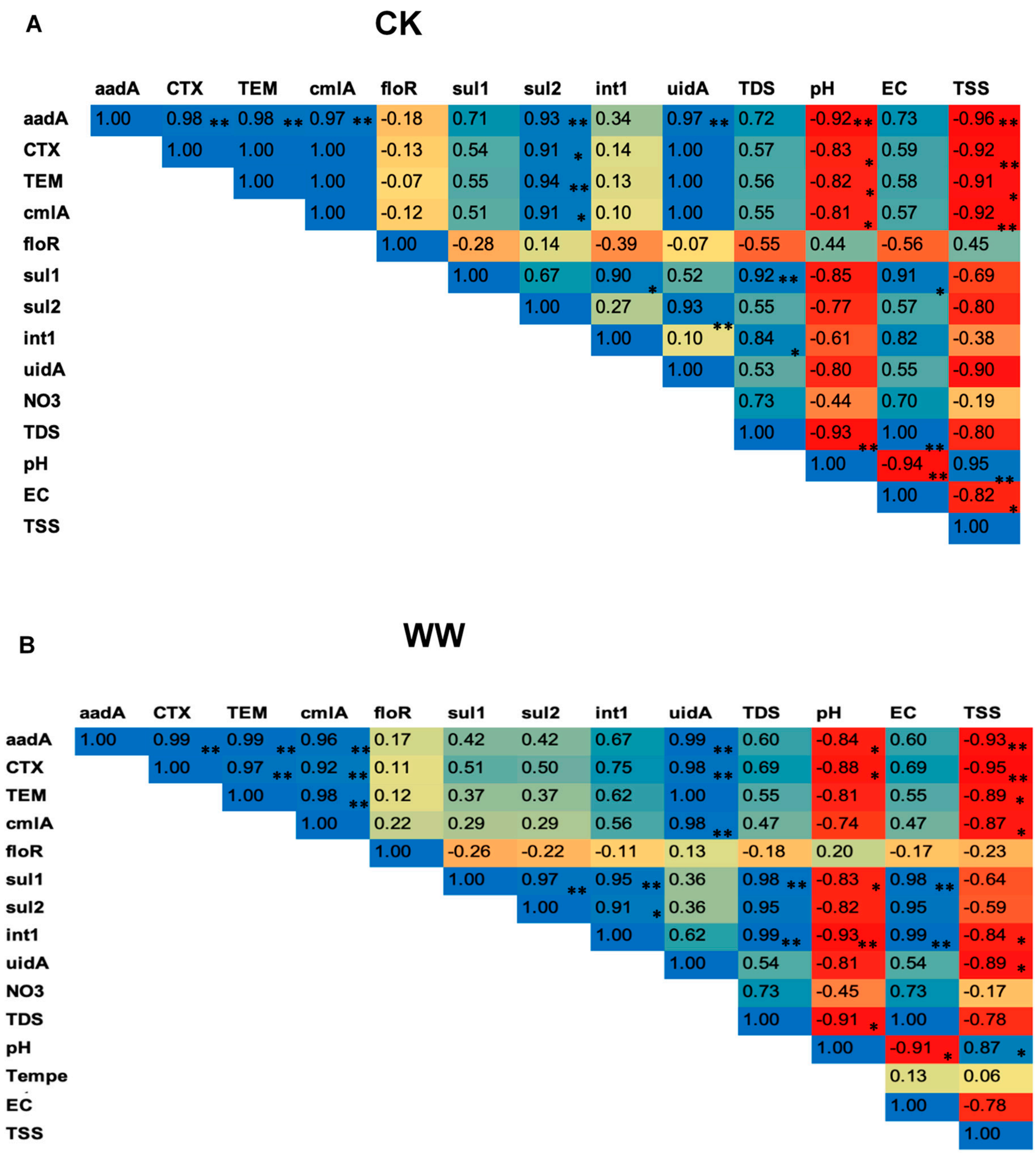

Figure S2. Correlation of the study genes with physicochemical water quality parameters in the multi-species cultures. **Correlation is significant at the 0.01 level (2-tailed); *Correlation is significant at the 0.05 level (2-tailed)
